# Occupational Exposure to Zoonotic Tuberculosis Caused by *Mycobacterium caprae*, Northern Greece, 2019

**DOI:** 10.3201/eid2707.204399

**Published:** 2021-07

**Authors:** Dimitrios Papaventsis, George Dougas, Ourania Kalkouni, Simona Karabela, Katerina Manika

**Affiliations:** Sotiria Chest Diseases Hospital, Athens, Greece (D. Papaventsis, S. Karabela);; National Public Health Organization, Athens (G. Dougas, O. Kalkouni);; G. Papanikolaou Hospital, Thessaloniki, Greece (K. Manika)

**Keywords:** *Mycobacterium bovis*, *Mycobacterium caprae*, tuberculosis, zoonoses, clinical laboratory techniques, public health surveillance, breeders, livestock, goats, Greece, tuberculosis and other mycobacteria

## Abstract

Pulmonary tuberculosis caused by *Mycobacterium caprae* was diagnosed in a 65-year-old goat breeder from northern Greece. This case represents a documented occupational transmission of *M. caprae* and highlights the importance of enhanced laboratory screening and increased surveillance for zoonotic tuberculosis control.

*Mycobacterium caprae*, a member of *Mycobacterium tuberculosis* complex (MTBC), causes caprine tuberculosis (TB) (*1*). *M. caprae* has been isolated mainly in continental Europe and infects animals and humans (*2*–*4*). Although clinically indistinguishable from *M. tuberculosis* or *M. bovis*, *M. caprae* causes nearly one third of *M. bovis*–associated TB cases (*5*). Bovine TB control and pasteurization have made zoonotic TB rare; however, occupational risk exists for livestock farmers, veterinarians, slaughterhouse workers, butchers, and other persons working in close contact with livestock.

In August 2019, a 65-year-old male goat breeder from northern Greece was admitted to the Aristotle University Pulmonology Department at G. Papanikolaou Hospital (Thessaloniki, Greece) for weight loss, productive cough, and fever <38.5^o^ C that was not responding to a 10-day regimen of cefuroxime. He was a frequent smoker, reported chronic alcohol abuse, and had no other medical history. He reported living in the goat shelter, occasionally consuming unpasteurized milk, and having no contact with or hunting practices involving other animal species. Laboratory tests showed normocytic anemia (hemoglobin 10.4 g/dL) and an erythrocyte sedimentation rate of 112 mm/h. Chest radiograph and computed tomography scans revealed upper lobe cavities and infiltrations, predominantly on the left side (Figure, panels A–C). A tuberculin skin test result was 12 mm in size. TB was suspected; sputum samples were sent for molecular and bacteriologic testing to the National Reference Laboratory for Mycobacteria at Sotiria Chest Diseases Hospital (Athens, Greece). Acid-fast stain results were positive and MTBC detected by using TRCReady-80 (Tosoh Corp., https://www.tosoh.com). We determined drug sensitivity on a strain grown on sodium pyruvate-LJ medium by Genotype MTBDRplus (Hain LifeScience, https://www.hain-lifescience.de) and on solid media. We documented sensitivity to first-line anti-TB drugs and pyrazinamide. Because of the patient’s occupation, laboratory investigation included zoonotic species. GenoType MTBC differentiated the isolate as *M. bovis* subspecies *caprae*.

**Figure:**
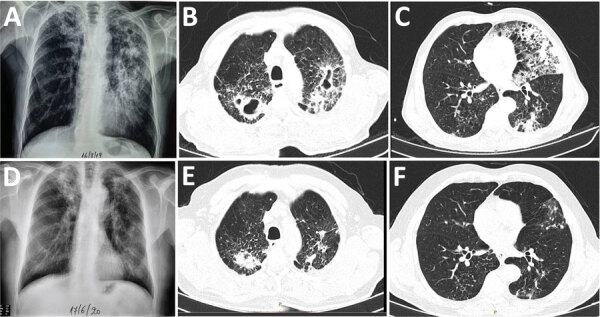
Comparison of chest radiographs and computed tomography scans before and after the end of treatment for a 65-year-old male goat breeder infected with *Mycobacterium caprae*, northern Greece, 2019. Multiple cavity infiltrations and opacities are shown on the chest radiographs (A) and the computed tomography scan (B, C), mainly in the left upper lobe. After treatment, significant improvement is shown by cavity closure and recession of opacities and infiltration on the chest radiograph (D) and on the chest computed tomography scans (E, F).

We used the optimized 24-loci Genoscreen MIRU-VNTR (mycobacterial interspersed repetitive unit–variable-number tandem-repeat) Typing Kit (Genoscreen, https://www.genoscreen.fr) on crude DNA, using 6 quadruplex PCR and fluorescent primers with capillary electrophoresis. Identification was performed with the MIRU-VNTR*plus* database (*6*). Because no match was detected after initial best-match analysis, we used a tree-based identification scheme by applying the UPGMA method and using the database reference strains. Identification was confirmed with a unique MIRU-VNTR pattern (M.caprae_Kilkis:255326322553434243231432) showing close phylogenetic match with *M. caprae* reference strains from central and eastern Europe and clear genetic distance from the Iberian cluster, mainly characterized by absence of spoligotype spacers 30–33 (*3*) (Appendix Figure).

The patient received a standard anti-TB regimen for 9 months and responded favorably. Cultures and acid-fast bacilli turned negative 10 days after treatment and were negative 2.5 months after treatment. Erythrocyte sedimentation rate dropped to 25 mm/h, and the patient gained 17 kg. Chest radiograph and computed tomography scans showed remarkable improvement (Figure, panels D–F). Contact tracing did not reveal further human cases. Veterinary investigation identified no tuberculin skin test reactors among >300 goats of the epidemiologically linked herd.

Zoonotic TB causes an estimated 147,000 new human cases and 12,500 deaths annually worldwide (*7*). Zoonotic TB is a reemerging and underrecognized infection in Europe; 170 confirmed human cases were reported in 2018 (0.05 cases/100,000 population) (*4*). Among countries not officially TB-free, Greece, Ireland, and Spain reported a plateaued prevalence of 2%–5% in cattle herds over the past decade (*4*). The actual global burden of *M. caprae* disease is further underestimated because of differences in laboratory capacity and lack of routine surveillance data. Zoonotic TB is a public health hazard, resulting in serious economic losses and having a substantial effect on poor and marginalized communities (*7*).

Contaminated food and airborne transmission pose considerable risks to persons in contact with infected animals or animal products (*7*). In this study, caprine TB was occupationally acquired by a goat farmer with a history of routine close contact with livestock. Pyrazinamide sensitivity and lung localization could have caused an initial misdiagnosis of *M. tuberculosis* infection. Since 2019, enhanced laboratory screening and surveillance for high-risk patients related to animal breeding have been used in Greece. Timely application of bacteriologic and genotyping techniques, less frequently used in routine laboratory investigation until recently, highlighted the role of *M. caprae* as a human pathogen. MIRU-VNTR revealed a unique isolate similar to patterns reported in animals in Eastern Europe and the Balkans (*3*,*8*). Data on *M. caprae* zoonotic TB in Greece are limited. A dairy goat farm outbreak in northern Greece was the first documented in 2005; *M. caprae* caused pneumonia among animals, and the mortality rate reached 92% (*9*). Further, Neonakis et al. (*10*) reported a human *M. bovis* isolate identified as *M. bovis* subsp. *caprae* in a GenoType MTBC assay evaluation study.

*M. caprae*, a recently identified separate MTBC species, should be carefully addressed nationally and internationally. Key risk populations in countries not officially TB-free and with sizeable goat populations must be identified, occupational history sought, testing capacity extended, and monitoring and reporting improved. A One Health approach that highlights interdependence of human and animal health sectors is needed to curb the spread of this pathogen.

Appendix Additional information about occupational exposure to zoonotic tuberculosis caused by *Mycobacterium caprae*, northern Greece, 2019.
