# Supplementary material for: Occupational Exposure to Zoonotic Tuberculosis Caused by Mycobacterium caprae, Northern Greece, 2019
Source: Emerg Infect Dis. 2021 Jul;27(7):1997–9. doi: 10.3201/eid2707.204399 (PMC8237881; doi:10.3201/eid2707.204399)
Supplement: Appendix — Additional information about occupational exposure to zoonotic tuberculosis caused by Mycobacterium caprae, northern Greece, 2019. [file 20-4399-Techapp-s1.pdf]

Article DOI: https://doi.org/10.3201/eid2707.204399

Occupational Exposure to Zoonotic Tuberculosis Caused by *Mycobacterium caprae*, Northern Greece, 2019

Appendix

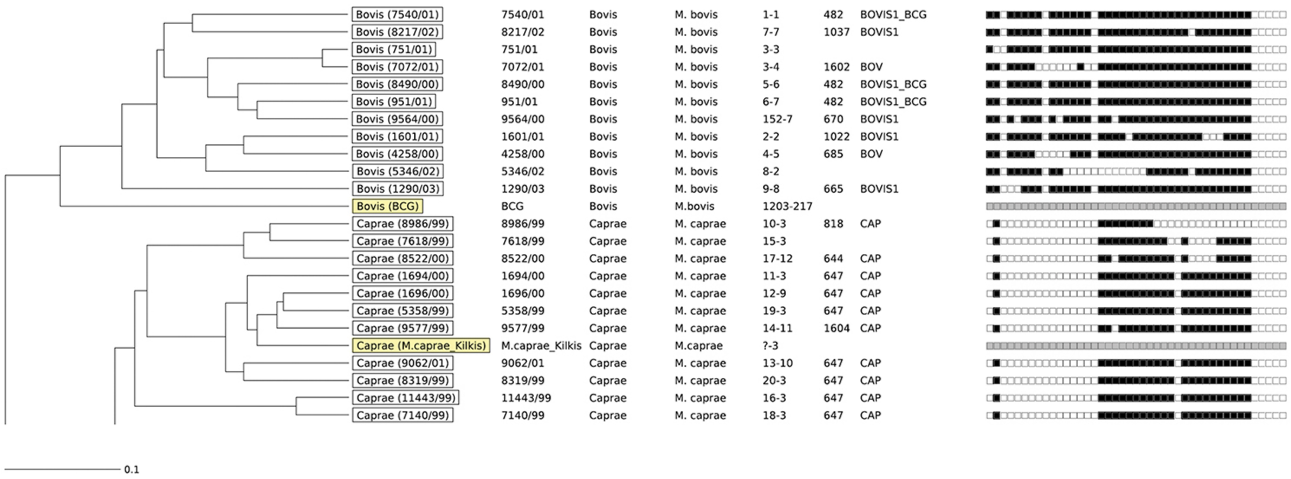

Appendix Figure. Phylogenic tree of a *Mycobacterium caprae* isolate from a 65-year-old goat breeder from Kilkis, northern Greece (M.caprae_Kilkis: MIRU-VNTR 24-loci pattern 255326322553434243231432). The tree was calculated and drawn by using the UPGMA method and utilizing the MIRU-VNTRplus database reference strains (*1*). VNTR patterns were first searched against the MIRU-VNTRplus database using a default stringent distance cut-off of 0.17, which corresponds to a tolerance of <u><</u>4 locus differences. Since no match was detected after the initial best-match analysis, a tree-based identification was used. Species, Lineage, MLVA MtbC15-9, SpolDB4 Type, and Spoligo pattern information has been included. The patient's isolate shows tight genetic clustering with *M. caprae* reference strains that dominate in central and eastern Europe (spoligotypes lacking spacers 1, 3 to 16, 28, and 39 to 43, BOV_4-CAPRAE, SIT647, SB0418). A clear genetic separation from the Iberian isolates, mainly characterized by the additional absence of spacers 30-33 (BOV-CAPRAE, SIT644/SB0157) has been demonstrated. The *M. bovis* BCG control is also depicted. Scale bar represents 10% sequence divergence. BCG, Bacillus Calmette–Guérin; MLVA, Multiple-locus variable-number tandem repeat analysis; VNTR, variable number of tandem DNA repeats

Reference

1. Allix-Béguec C, Harmsen D, Weniger T, Supply P, Niemann S. Evaluation and strategy for use of MIRU-VNTRplus, a multifunctional database for online analysis of genotyping data and phylogenetic identification of *Mycobacterium tuberculosis* complex isolates. J Clin Microbiol. 2008;46:2692–9. PubMed https://doi.org/10.1128/JCM.00540-08
